# Appraisal of anti-protozoan activity of nitroaromatic benzenesulfonamides inhibiting carbonic anhydrases from *Trypanosoma cruzi* and *Leishmania donovani*

**DOI:** 10.1080/14756366.2019.1626375

**Published:** 2019-06-20

**Authors:** Alessio Nocentini, Sameh M. Osman, Igor A. Rodrigues, Veronica S. Cardoso, Fatmah Ali S. Alasmary, Zeid AlOthman, Alane B. Vermelho, Paola Gratteri, Claudiu T. Supuran

**Affiliations:** a Department of Neuroscience, Psychology, Drug Research and Child's Health, Section of Pharmaceutical and Nutraceutical Sciences, University of Florence, Sesto Fiorentino, Italy;; b Department of Chemistry, College of Science, King Saud University, Riyadh, Saudi Arabia;; c Department of Natural Products and Food, School of Pharmacy, Federal University of Rio de Janeiro, Rio de Janeiro, Brazil;; d BIOINOVAR – Biotechnology Laboratories: Biocatalysis, Bioproducts and Bioenergy, Institute of Microbiology Paulo de Góes, Federal University of Rio de Janeiro, Rio de Janeiro, Brazil

**Keywords:** Carbonic anhydrase, Chagas disease, *Leishmania*, *Trypanosoma*, nitroaromatics

## Abstract

Chagas disease and leishmaniasis are neglected tropical disorders caused by the protozoans *Trypanosoma cruzi* and *Leishmania* spp. Carbonic anhydrases (CAs, EC 4.2.1.1) from these protozoans (α-TcCA and β-LdcCA) have been validated as promising targets for chemotherapic interventions. Many anti-protozoan agents, such as nitroimidazoles, nifurtimox, and benznidazole possess a nitro aromatic group in their structure which is crucial for their activity. As a continuation of our previous work on *N*-nitrosulfonamides as anti-protozoan agents, we investigated benzenesulfonamides bearing a nitro aromatic moiety against TcCA and LdcCA, observing selective inhibitions over human off-target CAs. Selected derivatives were assessed *in vitro* in different developmental stages of *T. cruzi* and *Leishmania* spp. A lack of significant growth inhibition has been found, which has been connected to the low permeability of this class of derivatives through cell membranes. Further strategies necessarily need to be designed for targeting Chagas disease and leishmaniasis with nitro-containing CA inhibitors.

## 1. Introduction

Chagas disease (American trypanosomiasis) and leishmaniasis are potentially life-threatening illnesses that have been included in the list of neglected tropical diseases (NTDs) by the World Health Organization (WHO). These infections belong to the vector-borne diseases affecting 20 million people and killing more than 50,000 every year and are caused by parasites of the kinetoplastida family (*Trypanosoma cruzi* and *Leishmania* sp.)^1^. Kissing bugs of the *Triatoma* and *Rhodnius* genera naturally transmit *T. cruzi* that is primarily diffused in Latin America. Chagas disease progresses by damaging organs in the cardiac, digestive, or nervous systems^1^. The bite of infected phlebotomines instead is the main cause of *Leishmania* transmission and potentially generates skin or visceral fatal damages. Leishmaniasis is the first-in-class NTD in terms of mortality and morbidity^1^.

To date, a limited arsenal of anti-protozoan agents is available for the treatment of these NTDs. These drugs are marked by high toxicity and limited efficacy, and resistance phenomena are constantly increasing worldwide^2–4^. The poor interest shown by the pharmaceutical industry in searching new effective drugs for NTDs treatment is related to high costs and expected low financial return. On the contrary, it should be considered a priority to find new approaches in the treatment of these parasitosis^2^^,^^5^. Large-scale analysis on the completely known genome sequence of both protozoans have recently provided the identification of new enzymatic targets^6^^,^^7^.

The enzymes carbonic anhydrases (CAs, EC 4.2.1.1) identified in these protozoans, TcCA in *T. cruzi* and LdcCA in *L. donovani* (a parasite from the *Leishmania* complex, causing visceral leishmaniasis) have recently been recognised as suitable targets to fight these infections^6^^,^^8^^,^^9^. CAs are natural catalysts that speed up the rate of CO_2_ conversion to bicarbonate and proton. This reaction was shown to be basic in the growth and virulence of pathogenic microorganisms^9^. TcCA and LdcCA were both cloned and characterised in 2013^10–12^. Many inhibitors of these isoforms have been identified, which represent potential anti-protozoan agents acting by a new mechanism of action which is probably devoid of cross-resistance to the existing drugs.

TcCA is an α-class enzyme that contains the three highly conserved histidines (His94, His96, and His119) coordinating to zinc ion in the enzyme active site, and glutamic acid (Glu 106) as the gate-keeping residue^10^. Measurement of the catalytic activity of TcCA in CO_2_ hydration showed a *k_cat_* of 1.21 × 10^6^ s^–1^, *K_m_* of 8.1 × 10^−3^ M and *k_cat_*/*K_m_* of 1.49 × 10^8^ M^–1^ s^–1^^10^. TcCA is inhibited in the nanomolar range by many CA inhibitory chemotypes such as aromatic/heterocyclic sulfonamides^10^^,^^13^^,^^14^, sulfamates^10^, thiols^10^, anions^15^, dithiocarbamates^15^, hydroxamates^16^, benzoxaboroles^17^, and N-nitrosulfonamides^18^. Thiols, hydroxamates, and N-nitrosulfonamides show *in vitro* anti-trypanosomal activity, deterring multiple phases in the life cycle of the pathogen^10^^,^^16^^,^^18^.

LdcCA is a β-class CA whose catalytic activity evaluation reported a *k_cat_* of 9.35 × 10^5^ s^–1^, *K_m_* of 15.8 × 10^−3^ M, and *k_cat_*/*K_m_* of 5.9 × 10^7^ M^–1^ s^–1^^12^. LdcCA was shown to be efficiently inhibited by sulfonamides, heterocyclic thiols, and N-nitrosulfonamides with nanomolar inhibition constants^12^^,^^18^. Some compounds of the two latter classes showed *in vitro* anti-leishmanial activity in preliminary assays, causing the reduction of the parasites growth and their death^12^^,^^18^.

*N*-Nitrosulfonamides have been designed by us based on the presence of the nitro group in the structure of many anti-protozoan agents, such as the nitroimidazoles, this moiety being pivotal for the drug mechanism of action^18^^,^^19^. For instance, nifurtimox and benznidazole (Bzn) have been the first effective drugs for treating acute-phase human Chagas infection, with the first being no longer available on the market because of undesirable side effects^6^. Considering that sulfonamides are the most effective CAIs known to date^20^, we first attached the nitro group on the sulfonamide itself, providing the *N*-nitro derivatives as a new chemotype exhibiting a selective inhibition of protozoan CAs over human ubiquitous isoforms^18^. As second design strategy, we report herein, consists in the incorporation of the nitro group on the benzene scaffold bearing the sulfonamide, driven by the aromatic character shown by the nitro moieties present in many anti-protozoan agents, mentioned above. A set of 3-nitrobenzenesulfonamide bearing a variety of substituents on the main scaffold has thus been reported. This set has been recently evaluated also for the inhibition of the human tumour-associated CA IX and XII over the ubiquitous CA I and II and for hypoxia-enhanced anti-proliferative activity on tumour cell lines^21^. In fact, nitroaromatic groups are subjected to bioreduction processes in hypoxic tissues, which can be exploited to selectively generate cytotoxins against tumour cells^21^. Here, the set of nitro-benzenesulfonamides has been screened for the inhibition of TcCA and LdcCA and the most effective derivatives were studied *in vitro* against different species of *Leishmania* and *T. cruzi*.

## 2. Materials and methods

### 2.1. Chemistry

The synthesis of 3-nitro-4-hydroxy-sulfonamides **4**–**24** was reported earlier by our group^21^.

### 2.2. Carbonic anhydrase inhibition

An Applied Photophysics stopped-flow instrument has been used for assaying the CA-catalysed CO_2_ hydration activity^22^. Phenol red (at a concentration of 0.2 mM) has been used as indicator, working at the absorbance maximum of 557 nm, with 20 mM Hepes (pH 7.5) as buffer, and 20 mM Na_2_SO_4_ (for maintaining constant the ionic strength), following the initial rates of the CA-catalysed CO_2_ hydration reaction for a period of 10–100 s. The CO_2_ concentrations ranged from 1.7 to 17 mM for the determination of the kinetic parameters and inhibition constants. For each inhibitor, at least six traces of the initial 5–10% of the reaction have been used for determining the initial velocity. The uncatalysed rates were determined in the same manner and subtracted from the total observed rates. Stock solutions of inhibitor (0.1 mM) were prepared in distilled-deionised water and dilutions up to 0.01 nM were done thereafter with the assay buffer. Inhibitor and enzyme solutions were preincubated together for 15 min at room temperature prior to assay, in order to allow for the formation of the E–I complex. The inhibition constants were obtained by nonlinear least-squares methods using PRISM 3 and the Cheng–Prusoff equation, as reported earlier, and represent the mean from at least three different determinations^23–26^. All CA isoforms were recombinant ones obtained in-house as reported earlier^27^^,^^28^.

### 2.3. Biological assays

#### 2.3.1. Cell cultures

##### 2.3.1.1. *Trypansoma cruzi* and *Leishmania* parasites cultures

Epimastigote forms of the *T. cruzi* clone Dm28c^29^ and *T. cruzi* Y^30^ strain were obtained from the Laboratory of Cellular Ultrastructure, FIOCRUZ. *L. infantum* MHOM/BR/1974/PP75 and *L. amazonensis* IFLA/BR/1967/PH8 were donated by the *Leishmania* Type Culture Collection (LTCC) of Oswaldo Cruz Institute/Fiocruz (Rio de Janeiro, Brazil). The parasites were maintained by weekly subcultures in PBHIL medium supplemented with 10% foetal bovine serum (FBS) at 28 °C^16^.

##### 2.3.1.2. RAW 264.7 macrophage cell line cultures

RAW 264.7 murine macrophages were obtained from the National Institute of Metrology, Quality and Technology (Instituto Nacional de Metrologia, Qualidade e Tecnologia, INMETRO, Rio de Janeiro, Brazil) and maintained in DMEM medium supplemented with 10% FBS at 37 °C in a 5% controlled CO_2_ atmosphere. Cell maintenance was performed every 48–72 h, time necessary for cells to achieve confluent monolayers.

#### 2.3.2. Inhibitory activity on epimastigotes of *Trypanosoma cruzi* and promastigotes of *Leishmania*

The evaluation of anti-parasites activity was performed in 96 well plates where the synthetic compounds were serially diluted in the PHBIL medium supplemented with 10% FBS in concentrations ranging from 2 to 400 µM. Then, parasites (1.8 × 10^6^) were added to each well and the plates incubated for 48 h at 28 °C. The experiment controls were: negative control (culture medium without parasite) and positive culture (culture medium with parasite). Benznidazole and amphotericin B (Amp) were used as reference drugs of *T. cruzi* and *Leishmania*, respectively. The minimum inhibitory concentration (MIC) for epimastigotes (*T. cruzi* DM28c and Y) and promastigotes (*L. amazonensis* and *L. infantum*) was performed using resazurin (125 µM) as an indicator of cellular metabolic function. MIC was determined as the lowest concentration of the inhibitor capable of inhibiting *in vitro* growth of the parasites by spectrophotometric analysis at 490 and 595^31^. The concentration of drug which reduces parasites number by 50% (IC_50_) was determined by regression analysis using Microsoft Excel 2013.

#### 2.3.3. Cytotoxicity assay in macrophages

Cytotoxicity was performed using tetrazolium dye (MTT) colorimetric assay. RAW 264.7 macrophages cells were harvested after confluent monolayer achievement^32^. The cells were washed twice with PBS and a cellular suspension of 10^6^ cells/ml was prepared in fresh DMEM culture medium. Aliquots of 100 µl of the cellular suspension were placed into polystyrene 96-well plates, and then incubated at 37 °C in a 5% CO_2_ atmosphere for 6 h in order to allow macrophage adherence. After this period, the adherent cells were subjected to treatment with several concentrations of the drugs (2–256 µM), and then incubated for additional 48 h. Finally, 20 µl of MTT solution (5 mg/ml) were added to each well and the plates incubated for 4 h. Macrophage viability was determined after formazan crystals solubilisation with DMSO followed by the absorbance measurement at 570 nm using a SpectraMax M5 spectrophotometer (Molecular Devices, Sunnyvale, CA).

#### 2.3.4. Determination of selectivity index

The selectivity index (SI) of tested drugs was calculated as a ratio of RAW 264.7 macrophages CC_50_ to parasites IC_50_. Benznidazole (Sigma-Aldrich, Milan, Italy) and Amp were used as reference drugs.

## 3. Results and discussion

### 3.1. Chemistry

**Scheme 1.**
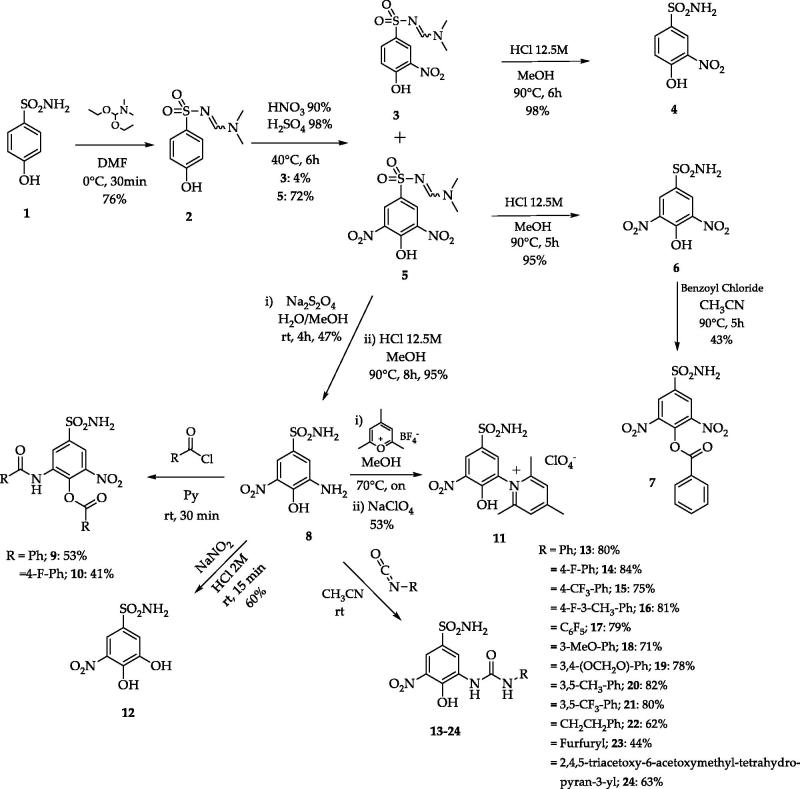
Synthetic routes to 3-nitrobenzenesulfonamides **3**–**24**^21^.

**Scheme 2.**
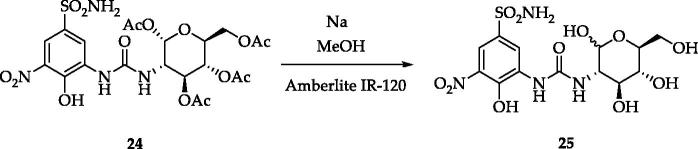
Synthesis of derivative **25**.

A set of variably substituted 3-nitrobenzenesulfonamides was prepared starting from 4-hydrobenzenesulfonamide **1**^21^. The sulfonamide moiety was protected (compound **2**) to avoid decomposition to sulfonic acid that occurs in the nitrating conditions. Both mono- and di-nitro derivatives were obtained in different yields and deprotected in acidic media (compounds **4** and **6**). 3,5-Dinitro compound **6** was benzoylated to afford **7**. A key intermediate, 3-amino-4-hydroxy-5-nitro-benzenesulfonamide **8** was achieved by reduction of a unique nitro group of **5** with Na_2_S_2_O_4_ and sequential sulfonamide deprotection in acidic media. Intermediate **8** was subjected to several functionalisation reactions. Acylation reactions produced the di-benzoyl compounds **9** and **10**. The pyridinium salt **11** was prepared by the reaction of **8** with the proper pyrylium salt. The light-sensitive derivative **12** was obtained by diazonium salt formation and N_2_ release in aqueous NaNO_2_^21^. A set of ureas (**13**–**24**) was prepared by the reaction of **8** with commercially available isocyanates, in addition to the freshly prepared one obtained from 1,3,4,6-tetra-*O*-acetyl-glucosamine^21^. Compound **24** was de-acetylated with sodium methoxide to give the glycoside **25** (Schemes 1 and 2).

### 3.2. Carbonic anhydrase inhibition

The TcCA and LdcCA inhibitory profiles of compounds **4**–**25** were evaluated by applying a stopped flow carbon dioxide hydrase assay^22^ in comparison to **AAZ** as standard CAI and compared to those against the human off-target CA I and II. The following SAR can be built from the inhibition data shown in Table 1.

**Table 1.** Inhibition data of TcCA, LdCA and human CA I and II with sulfonamides reported here and the standard inhibitor acetazolamide (AAZ) by a stopped flow CO_2_ hydrase assay.

Compound	R	*K*_I_ (μM)^a^			
		TcCA	LdCA	hCA I	hCA II
**4**		0.08	0.21	0.91	0.24
**6**		0.16	0.34	4.35	0.18
**7**		2.52	4.68	4.79	0.84
**8**		0.24	0.46	6.18	0.61
**9**	C_6_H_5_	3.54	3.87	1.38	0.39
**10**	4-F-C_6_H_4_	4.79	8.49	2.92	0.46
**11**		10.7	6.57	>50	1.81
**12**		0.11	0.39	6.21	0.64
**13**	C_6_H_5_	0.32	1.06	>50	2.78
**14**	4-F-C_6_H_4_	0.46	0.98	5.39	0.53
**15**	4-CF_3_-C_6_H_4_	0.51	2.34	5.20	0.24
**16**	4-F-3-CH_3_-C_6_H_3_	0.38	2.96	7.58	0.21
**17**	C_6_F_5_	0.28	1.36	0.69	0.27
**18**	3-CH_3_O-C_6_H_4_	0.91	0.95	8.21	5.15
**19**	3,4-(OCH_2_O)-C_6_H_3_	1.02	2.03	>50	4.33
**20**	3,5-CH_3_-C_6_H_3_	0.69	1.86	8.33	0.45
**21**	3,5-CF_3_-C_6_H_3_	1.35	3.65	5.99	1.72
**22**	CH_2_CH_2_C_6_H_5_	0.74	0.86	9.29	3.08
**23**	CH_2_-(2-furyl)	0.41	1.02	>50	2.53
**24**	2,4,5-triacetoxy-6-acetoxymethyl-tetrahydro-pyran-3-yl	2.47	3.64	5.67	1.89
**25**	2,4,5-trihydroxy-6-hydroxymethyl-tetrahydro-pyran-3-yl	2.14	2.97	4.92	0.86
**AAZ**	–	0.06	0.09	0.25	0.012

a Mean from three different assays, by a stopped flow technique (errors were in the range of ±5–10% of the reported values).

TcCA was effectively inhibited by most 3-nitrobenzenesulfonamides investigated here. Inhibition constants (*K*_I_s) span in medium nanomolar to low micromolar range between 0.08 and 10.7 µM. The derivative showing the lowest steric hindrance, namely **4**, acts as the most potent TcCA inhibitor with a *K*_I_ of 80 nM. The incorporation of a nitro, amino or hydroxy moiety in position 5 of compound **4** as in **6**, **8**, and **11** lowered the inhibition efficacy to 160, 240, and 110 nM, respectively. Lowering of inhibition potency was observed by benzoylation of **6** as in **7** (*K*_I_ of 2.5 µM) or dybenzoylation of **8** as in **9** and **10** (*K*_I_s of 3.5 and 4.8 µM). Hence, enhancement of steric hindrance at position 4 has a deleterious effect on the compounds binding to TcCA. Incorporation of a positively charged moiety, such as pyridinium at position 5 in compound **11**, caused an evident drop of inhibitory efficacy (*K*_I_ of 10.7 µM). Within the set of ureas, the unsubstituted phenyl derivative **13** and the pentafluorinated one **17** showed the most effective inhibition, with *K*_I_s of 0.32 and 0.28 µM. All other substitutions on the ureido-aromatic ring negatively affect the inhibition to 0.35–1.35 µM. Insertion of aliphatic linkers between the urea and the outer aromatic portion also has a negative outcome on the *K*_I_s of **22** and **23**, which increase to 0.74 and 0.4 µM. The glycosidic derivatives **24** and **25** are micromolar TcCA inhibitors with *K*_I_s of 2.47 and 2.14 µM.

LdcCA inhibition profiles show analogies with those against TcCA. Again, the simplest derivatives **4**, **6**, **8**, and **12** act as the best LdcCA inhibitors with *K*_I_s of 0.21, 0.34, 0.46, and 0.39 µM, respectively. Benzoylation of the hydroxy moiety at position 4 markedly reduced the LdcCA inhibitory properties of **7**, **9**, and **10** (*K*_I_s of 4.68, 3.87, and 8.49 µM) as well as incorporation of the charged pyridinium portion as in **11** (*K*_I_ of 6.57 µM). Ureido derivatives **13**–**25** inhibited LdcCA in a rather flat range spanning from 0.86 to 3.65 µM.

As a general trend, most compounds were more effective against TcCA than CA I, with an SI from 2 to >150, with the exception of **9** and **10** (Table 2). On the other hand, only few derivatives (**8**, **12**, **13**, **18**, **19**, **22**, and **23**) inhibited TcCA more efficiently than CA II, with SI spanning between 2.5 and 6. LdcCA was found to be better inhibited than hCA I by most compound, though the SIs were lower than those TcCA/CA I, and spanned in the range of 2–50. Most compounds inhibited CA II better than LdcCA. All compounds inhibited the screened isoforms worse than the standard **AAZ**, but the latter did not show selectivity for the target TcCA and LdcCA compared to the ubiquitous hCAs (Table 2).

**Table 2.** Selectivity index (SI) for target protozoan CAs over hCA I and II.

Compound	SI (*K*_I1_/*K*_I2_)			
	CA I/TcCA	CA II/TcCA	CA I/LdcCA	CA II/LdcCA
**4**	11.4	3.0	4.3	1.1
**6**	27.2	1.1	12.8	0.5
**7**	1.9	0.3	1.0	0.2
**8**	25.8	2.5	13.4	1.3
**9**	0.4	0.1	0.4	0.1
**10**	0.6	0.1	0.3	0.1
**11**	>4.7	0.2	>7.6	0.3
**12**	56.5	5.8	15.9	1.6
**13**	>156	8.7	>47.2	2.6
**14**	11.7	1.2	5.5	0.5
**15**	10.2	0.4	2.2	0.1
**16**	19.9	0.6	2.6	0.1
**17**	2.5	1.0	0.5	0.2
**18**	9.0	5.7	8.6	5.4
**19**	>49.0	4.2	>24.6	2.1
**20**	12.1	0.7	4.5	0.2
**21**	4.4	1.3	1.6	0.5
**22**	12.6	4.2	10.8	3.6
**23**	>125	6.3	>49.0	2.5
**24**	2.3	0.8	1.6	0.5
**25**	2.3	0.4	1.7	0.3

### 3.3. Anti-protozoan activity

#### 3.3.1. *Trypanosoma cruzi* strain DM28c and Y

Ten selected derivatives bearing different substituents at the 3-nitrobenzenesulfonamide scaffold (**4**, **6**, **8**, **10**, **11**, **17**, **18**, **19**, **21**, and **24**) were screened for their inhibition activity different species of *Leishmania* and *Trypanosoma cruzi*.

The MIC and IC_50_ values against *T. cruzi* epimastigote forms of these compounds are shown in Table 3. The experiments showed that no compounds significantly affect the growth of the pathogen below 256 µM. The reference drug Bzn showed IC_50_ values against Dm28c clone and Y strains of 16.56 ± 1.51 and 6.54 ± 1.82 µM, respectively. The assessment of the toxicity of the selected 3-nitrobenzensulfonamides for Raw 267.4 macrophages cells showed that most derivatives were less toxic than Bzn (CC_50_ of 115.14 ± 9.48 µM) with CC_50_ above 172.65 ± 10.44 µM. Compounds **4** and **6** showed instead comparable toxicity with Bnz with CC_50_ values of 97.65 ± 11.13 and 100.21 ± 17.27 µM.

**Table 3.** Minimum inhibitory concentration (MIC), IC_50_ values derived from growth inhibition assays of *T. cruzi* Dm 28c and Y, determination of cytotoxicity (CC_50_), selectivity index (SI_50_) of compounds **4**, **6**, **8**, **10**, **11**, **17**, **18**, **19**, **21**, and **24**.

Compound	*T. cruzi* Dm 28c			*T. cruzi* Y			CC_50_ (µM)^d^
	MIC (µM)^a^	IC_50_ (µM)^b^	SI^c^	MIC (µM)^a^	IC_50_ (µM)^b^	SI^c^	
**4**	>256	n.d.	n.d.	>256	n.d.	n.d.	97.65 ± 11.13
**6**	>256	n.d.	n.d.	>256	n.d.	n.d.	100.21 ± 17.27
**8**	>256	n.d.	n.d.	>256	n.d.	n.d.	>256
**10**	>256	n.d.	n.d.	>256	n.d.	n.d.	179.93 ± 21.31
**11**	>256	n.d.	n.d.	>256	n.d.	n.d.	>256
**17**	>256	n.d.	n.d.	>256	n.d.	n.d.	>256
**18**	>256	n.d.	n.d.	>256	n.d.	n.d.	>256
**19**	>256	n.d.	n.d.	>256	n.d.	n.d.	172.65 ± 10.44
**21**	>256	n.d.	n.d.	>256	n.d.	n.d.	>256
**24**	>256	n.d.	n.d.	>256	n.d.	n.d.	>256
**Bzn**	32	16.56 ± 1.51	6.54 ± 1.82	32	18.45 ± 0.32	6.168 ± 0.81	115.14 ± 9.48

a Minimum inhibitory concentration.

b µM **–** concentration which reduced the proliferation of epimastigotes by 50%.

c Selectivity index of 50% = CC_50_/IC_50_.

d µM – concentration cytotoxic which reduced 50% of RAW 267.4 cells.

#### 3.3.2. *L. amazonensis* and *L. infantum*

The MIC and IC_50_ values of the selected compounds against two *Leishmania* species are shown in Table 4. The experiments on *L. amazonensis* and *L. infantum* strains did not show MIC values below 400 µM. Despite the compounds showed micromolar inhibition of LdcCA, their efficacy turned out to be insignificant when translated *in vitro* against the pathogen cell cultures. The reference drug Amp exhibited IC_50_ values against the two strains of 1.65 ± 0.28 and 1.77 ± 0.35 µM, respectively. The tested sulfonamides showed anyhow remarkably minor toxicity for Raw 267.4 macrophages cells compared to Amp, that has a CC_50_ of 1 µM against both strains.

**Table 4.** Minimum inhibitory concentration (MIC), IC_50_ values derived from growth inhibition assays of *L. amazonensis* and *L. infantum*, determination of cytotoxicity (CC_50_) and selectivity index (SI_50_) of compounds **4**, **6**, **8**, **10**, **11**, **17**, **18**, **19**, **21**, and **24**.

Compound	*L. amazonensis*			*L. infantum*			CC_50_ (µM)^d^
	MIC (µM)^a^	IC_50_ (µM)^b^	SI^c^	MIC (µM)^a^	IC_50_ (µM)^b^	SI^c^	
**4**	>400	n.d.	n.d.	>400	n.d.	n.d.	97.65 ± 11.13
**6**	>400	n.d.	n.d.	>400	n.d.	n.d.	100.21 ± 17.27
**8**	>400	n.d.	n.d.	>400	n.d.	n.d.	>256
**10**	>400	n.d.	n.d.	>400	n.d.	n.d.	179.93 ± 21.31
**11**	>400	n.d.	n.d.	>400	n.d.	n.d.	>256
**17**	>400	n.d.	n.d.	>400	n.d.	n.d.	>256
**18**	>400	n.d.	n.d.	>400	n.d.	n.d.	>256
**19**	>400	n.d.	n.d.	>400	n.d.	n.d.	172.65 ± 10.44
**21**	>400	n.d.	n.d.	>400	n.d.	n.d.	>256
**24**	>400	n.d.	n.d.	>400	n.d.	n.d.	>256
**Amp**	8	1.65 ± 0.28	0.60	8	1.77 ± 0.35	0.56	1.0

a MIC – minimum inhibitory concentration.

b IC_50_ µM – concentration which reduced the number of promastigotes by 50%.

c SI_50_–selectivity index of 50% = CC_50_/IC_50_.

d CC_50_ µM – cytotoxic concentration which reduced 50% of RAW 267.4 viability.

Unfortunately, the tested 3-nitrobenzenesulfonamides turned out to be ineffective *in vitro* against strains of *T. cruzi* and *Leishmania*. The lack of activity is not a totally new issue in the field of sulfonamide CAIs against pathogens. For instance, some sulfonamide derivatives demonstrated remarkable *in vitro* efficacy in inhibiting the β-CA from the yeast *Malassezia globosa*, arousing anyhow complications *in vivo* because of permeability problems through biological membranes^33^.

In the context of *T. cruzi* and *Leishmania*, some previously tested sulfonamides showed an absence of anti-protozoan efficacy, which has been related to the lack of permeability through the biological membranes of the pathogen^34–36^. Hence, a formulation of such sulfonamides in nano-emulsions (NEs) of clove oil was attempted to enhance their bioavailability and penetrability through membranes^34^^,^^35^. The drugs–NEs formulations potently inhibited the growth of *T. cruzi* and *Leishmania in vitro*, with a huge increase of efficacy over the sulfonamide CAI alone. NEs turned out as a novel vehicle for the delivery of such hydrophilic drugs.

Indeed, it should be noted that 3-nitro-4-hydroxybenzenesulfonamides reported here are even more hydrophilic, which can cause difficulties for the compounds to cross the protozoa cell membrane and inhibit the cytoplasmatic CAs or exert further actions due to the nitro group. Hence, formulation to enhance the compounds bioavailability, such as NEs, is being prepared to evaluate the real anti-protozoan efficacy of these set of nitroaromatic CAIs.

## 4. Conclusions

We proposed here nitroaromatic sulfonamides for the treatment of Chagas disease and leishmaniasis based on CA inhibition. As a continuation of a previous work of us on N-nitrosulfonamides as anti-protozoan agents, we studied here benzenesulfonamides (**4**–**24**) bearing a nitro moiety on the aromatic scaffold against TcCA from *T. cruzi*, responsible of Chagas disease, and LdcCA from *Leishmania* spp. The compounds reported valuable micromolar inhibition of these two enzymes, in some cases even selective for the target CAs over the human ubiquitous CA I and II. Unfortunately, a selected set of such derivatives tested *in vitro* against multiple strains of *T. cruzi* and *Leishmania* did not produce growth inhibition of the parasites. The lack of anti-protozoan efficacy of sulfonamide type derivatives had been already reported by us and justified by low permeability of this class of derivatives through the cell membranes. The use of carriers such as nanoemulsions allowed to overcome this issue. The application of this approach has been being carried out for 3-nitrosulfonamides **4**–**24** to elucidate whether the combination of CA inhibition and further anti-protozoan actions related to the nitro group could be a winning anti-infective strategy.
